# Microfluidic Systems Applied in Solid-State Nanopore Sensors

**DOI:** 10.3390/mi11030332

**Published:** 2020-03-23

**Authors:** Jiye Fu, Linlin Wu, Yi Qiao, Jing Tu, Zuhong Lu

**Affiliations:** State Key Laboratory of Bioelectronics, School of Biological Science and Medical Engineering, Southeast University, Nanjing 210096, China; candidefu@163.com (J.F.); 220181825@seu.edu.cn (L.W.); qiaoyilll@163.com (Y.Q.)

**Keywords:** solid-state nanopore, microfluidic system, multichannel, optical detection, integration

## Abstract

Microfluidic system, as a kind of miniature integrated operating platform, has been applied to solid-state nanopore sensors after many years of experimental study. In the process of introducing microfluidic into solid-state nanopore sensors, many novel device structures are designed due to the abundance of analytes and the diversity of detection methods. Here we review the fundamental setup of nanopore-based microfluidic systems and the developments and advancements that have been taking place in the field. The microfluidic systems with a multichannel strategy to elevate the throughput and efficiency of nanopore sensors are then presented. Multifunctional detection represented by optical-electrical detection, which is realized by microfluidic integration, is also described. A high integration microfluidic system with nanopore is further discussed, which shows the prototype of commercialization.

## 1. Introduction

Nanopore sensors normally rely on the continuous electrical detection from both sides of the pore, which have been exploited to the application of DNA sequencing [1,2,3], single-molecule detection [4,5,6], molecular morphology analysis [7,8,9,10], drug screening [11] and even seawater desalination [12,13] over the past two decades. Nanopore can be divided into biological nanopore formed by embedding proteins with a pore in lipid bilayer membranes and solid-state nanopore fabricated on ultrathin solid-state membranes. Biological nanopores have good biocompatibility and a low detection noise, which makes them achieve greater accuracy currently in single-molecule sensing compared to solid-state nanopores. Furthermore, Oxford Nanopore Technology Ltd. developed a DNA sequencer (MinION) by biological nanopores, which marked the systematization and commercialization of biological nanopores [14,15]. Meanwhile, biological nanopores have their drawbacks of fragility of lipid bilayer membrane and fixed nanopore size, which limit their applications in larger-sized molecule detection.

In addition to biological nanopores, solid-state nanopores with tunable size, great stability, the extendibility of chemical modification and environmental tolerance, show potential in single-molecule detection [16]. For a typical solid-state nanopore device, the chip with nanopore separates two electrolyte solutions, and ionic current is evoked and measured by electrodes immersed in the electrolyte solutions. Many simple and easy devices were utilized for solid-state nanopore detection in the early research stage. Wharton and coworkers demonstrated a biosensor in which a conical nanopore was fabricated on a polyethylene terephthalate (PET) membrane [17]. Centimeter-level space was reserved for the solution chamber and a clamping screw was used to fix the nanopore chip. Si and coworkers used two epoxy blocks with complementary sliding tracks to immobilize the nanopore chip [18]. A round rubber washer was adopted on both sides to protect the chip. The electrolyte solution was directly injected into the epoxy pool by an injection syringe. Nevertheless, because of the bad sealing status, substances in the external environment can enter the electrolyte and affected the efficiency of detection. The large space between the nanopore chip inevitably led to the waste of samples. What’s more, the position of the electrodes might deviate every time the device was assembled, which is not conducive to the modeling of the detection system and the analysis of the ionic current data.

Microfluidic system, the technology involved in systems that use micro-channels to process or manipulate tiny fluids, shows excellent compatibility with nanopore sensors. Micro-scale flow channels and electrolyte solution chambers effectively reduce sample loss and consequently improve the detection efficiency. Changing from open inlet to a pair of inlet and outlet with great contact improves the tightness and cleanliness of the solution environment, as well as the freshness and fluidity of samples. Fixed and embedded microelectrodes reduce system noise and the chance of exposure to the air, which also provide better conditions to model the detection system. Chemical modification of the microfluidic system enhances the acquisition of analyte molecules and the specificity of their signals. Moreover, the powerful integration capabilities of the microfluidic system provide the potential for nanopore parallel detection and multifunctional detection, which expands the application field of nanopore biosensors.

This review first describes the fundamental configuration of a device combining nanopore and microfluidic systems. Some improvements to this structure will also be mentioned as the possible developing directions. The following section of this review is devoted to the endeavor in improving the detection efficiency of nanopore sensors by introducing the multichannel structure to the microfluidic system. Comparing with the single-channel structure, the design of the multichannel structure is more efficient and more user-friendly. The third section is dedicated to the nanopore multifunctional detection, in which optical detection was specifically discussed for its potential of realizing parallel nanopore detection without deploying mass of electrodes. The wider integrated microfluidic system is then mentioned. We end with a short discussion and some personal perspectives on this field.

## 2. Fundamental Configuration of a Nanopore-Based Microfluidic System

**Figure 1:**
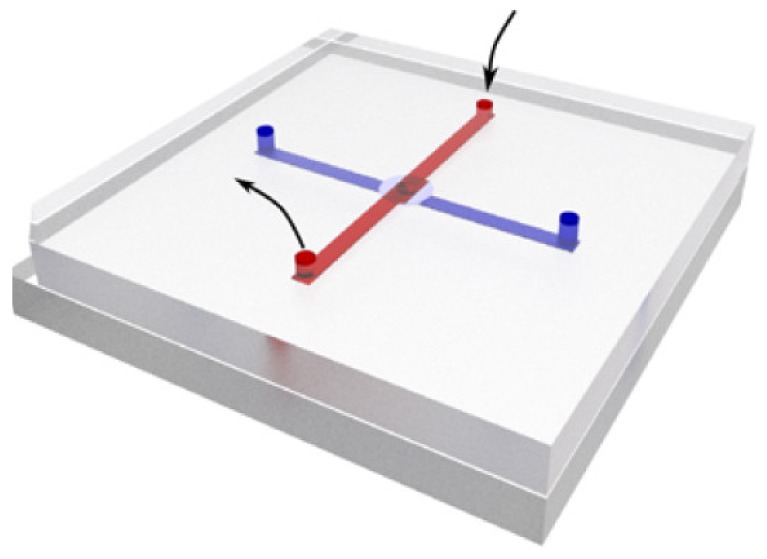
Schematic of fundamental configuration for nanopore-based microfluidic systems. A chip with one or an array of nanopores is clamped by two substrates with microchannels. Both sides of the chip have an inlet and outlet for electrolyte solutions. A pair of electrodes is applied in two channels and the nanopore is the only access for the ionic current and electrolyte solutions. Reprinted with permission from [27].

Nanopore sensor is typically a chamber with an insulating ultrathin membrane separating two parts of electrolyte, on which one or arrays of nano-sized holes are drilled by focused beams [19,20,21,22] or controlled dielectric breakdown [23,24]. Electrodes are put into both sides of electrolyte solutions to real-time monitor the ionic current across the hole. This brief structure indicates the basic requirements of the microfluidic system. Firstly, the inlet and outlet for the electrolyte solution are needed on both sides of the membrane to make sure the nanopore can be infiltrated and the two chambers can be connected electrically. Secondly, electrodes should be placed in direct contact with the electrolyte so that ionic current information across nanopore can be acquired in time. To get better performance of data analysis, the distance between two electrodes should be fixed and quantified. Figure 1 shows the schematic diagram of the fundamental configuration of the nanopore-based microfluidic system.

As early as 2003, a prototype of a nanopore-based microfluidic system, in which upper and bottom flow cell were linked by nanometer-diameter capillaries on the nuclear track-etched polycarbonate membrane, was studied by Kuo and coworkers [25]. In 2008, Zhou and coworkers studied the unique ion and molecule transport characteristics in the nanopore integrated microfluidic system [26]. They found that ion depletion and sample stacking were independent with the formation of double-layer overlap and excess surface charge within the pore could arise transport anisotropies. This work provided important theoretical reference for the design of channel size, the scheme of channel surface modification and the interpretation of nanopore data.

**Figure 2:**
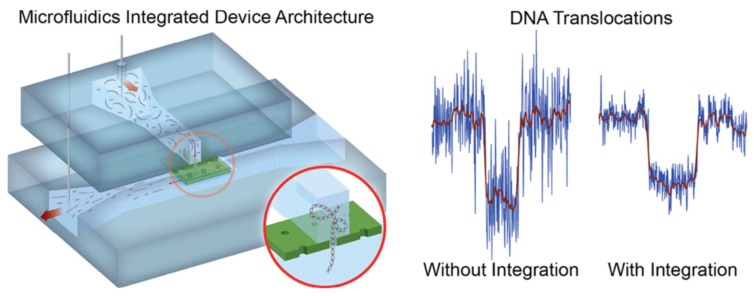
Schematic of microfluidic integrated nanopore device and the signal contrast with and without integrating. (**left**) Schematic view of microfluidic integrated nanopore device. This device is made up of two polydimethylsiloxane (PDMS) substrates, where perpendicular microchannel were located at and a 50 nm SiN_x_ membrane with square nanopore arrays. The pitch of the nanopore array was set to equal the microchannel width to ensure only one pore was registered between two microchannels. (**right**) The reduction in noise and enhancement in signal-to-ratio were achieved by integrating the microfluidic system to the nanopore sensor. Reprinted with permission from [28].

In the past two decades, this simple configuration integrating solid-state nanopores with microfluidic devices has been used in many research laboratories. Jain and coworkers demonstrated integration of nanopore and microfluidic systems by transferring the printing of suspended membranes (Figure 2) [28]. The SiN_x_ membrane used in this work was firstly drilled by a focus ion beam to form a nanopore array, then deposited with Al_2_O_3_ and SiO_2_, and finally detached from the Si substrate. This ultrathin membrane was bonded and clamped by two polydimethylsiloxane (PDMS) substrates with perpendicular microchannel and only one nanopore on the array could connect the upper and bottom channels by regulating the space between adjacent nanopores in the array. They successfully detected single DNA molecules with a significant reduction in the noise by using this microfluidic-integrated nanopore system compared with nonintegrated devices, which was clearly shown in the right of Figure 2.

**Figure 3:**
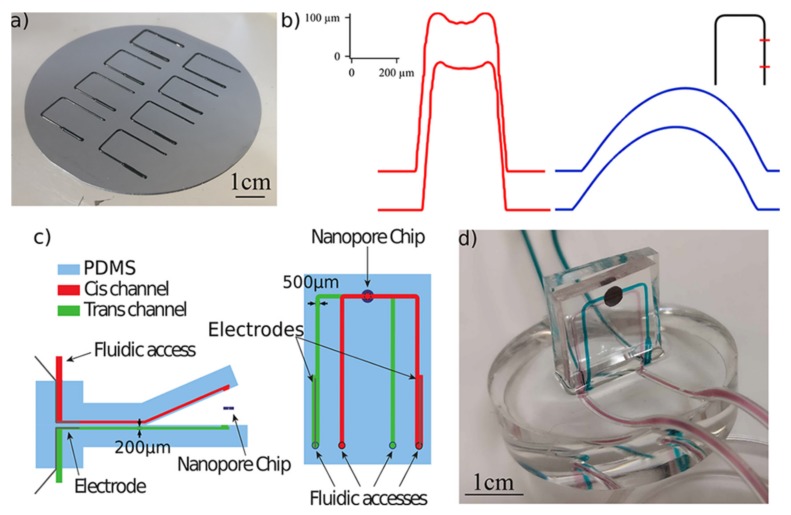
The PDMS-based microfluidic integrated system for efficient and convenient nanopore sensing. (**a**) Photograph of the PDMS microfluidic circuit mold on 3 in. Si wafer. Four pairs of flow cells can be fabricated and assembled into four devices in a single process. (**b**) Outlines of a 3D-printed mold before (red) and after (blue) melting. Uniformity of the printed parts is confirmed by two parallel controls and the locations are approximately exhibited by red marks on the inset black line. (**c**) Side and top schematic of the system. (**d**) Photograph of the system after mounting a nanopore chip. Reprinted with permission from [29].

Roman and coworkers focus on the economy and reusability of nanopore-based microfluidic devices [27,29]. They adopted 3D-printer technology in their works to manufacture the mold of the PDMS microfluidic circuit for saving time and money. Three-layer structure was employed in their system, which was shown in Figure 3c. PEG (Poly-ethylene glycol) chains were used to functionalize the microfluidic channel and the nanopore chip, and by controlling the density and length of PEG chains, the apparent radius and ionic conductance could be regulated easily. The PEG conformation, formed as an ideal polymer brush or a cigar-like shape, could be determined by an experiment of mimicking protein interaction with urea. For instance, the conductivity ratio of the PEG decorated pore maintained at 0.96 ± 0.07 in 10 mM KCl, while that in 10 mM LiCl, increased rapidly with the increase of the urea concentration. However, in a high concentration buffer (0.5 M), this ratio was steady. Convenience of disassembly and cleaning, avoidance of external contamination were also the advantages of this microfluidic system. Using the fundamental microfluidic setup, Leong and coworkers made an overall study of the influence of capacitance on the temporal resolution of the nanopore sensor by polymer coating [30]. The front-coated method which directly lies on the freestanding membrane and back-coated method that coated on the outer Si substrate were both tested. When the polymer layer was bigger than 12 mm, a strong suppression of noise could happen. Both of the methods had a delay on the resistive pulse, which were 145 and 149 μm respectively. Consequently, the back cover structure with the equivalent effect of noise suppression was recommended for its relatively small corresponding delay.

The synergistic effect of flow velocity and drift velocity exist in solid-state nanopore integrated microfluidic devices, which is a universal task for all nanopore-microfluidic devices, was presented by Guzel and coworkers [31]. The author gave the DNA capture regime as three parts: the diffusion-limited regime, transitional regime, and flow-limited regime, and they had different regions of action, which are determined by the location of the distance from the nanopore (within 500 nm or not), the driven speed of the electrical field and microfluidic flow. Although this work is at the early stage, but this research direction is important to the molecular dynamics and design of nanopore integrated microfluidics.

These microfluidic systems based on simple channel structure emphasized the best fit between microfluidic and nanopore systems, and minimized the interference of microfluidic to nanopore sensors and tried to provide a most primitive and ideal detection environment except the necessary elements. Because of the absence of complex channel and electrode structure, the reusability and convenience of the whole system have become the main direction of microfluidic systems’ design.

## 3. A Multichannel Structure for Detection Efficiency

### 3.1. Multi-Electrode for Multi-Channel Strategy

The nanopore sensors are based on a stochastic nature of single-molecule detection, so that means many individual translocating events should be recorded to get statistically significant data for a certain sample. In addition, the translocation speed of a single molecule should be controlled under the detection line of the system so that the translocation events can be observed as many as possible. These two factors result in low detection efficiency of nanopore sensors. To get a balance between the detection efficiency and precision, the multiplexed detection from an array of nanopores can considerably speed up detection, and simultaneously reduce the demand of the concentration of samples. However, it is unrealistic for one pair of electrodes to detect multi-channel signals, especially when these signals are originated from such small holes.

To solve this problem, the microfluidic system was adapted to the nanopore detection, by means of introducing fluid wells connected to electrode arrays. This idea has been experimental verification by Osaki, Suzuki and coworkers in biological nanopores [32,33,34]. They developed a microarray system that enables simultaneous monitoring of multiple ionic currents through transmembrane α-hemolysin nanopores arrayed at bilayer lipid membranes. Baaken and coworkers also designed a biological nanopore microarray to parallel single-molecule analysis electrically with high-resolution and used a polymer mass spectrometry experiment as a benchmark to prove the accuracy of this system [35]. Similar multi-channel structures are used in many works on biological nanopores subsequently [36,37,38].

**Figure 4:**
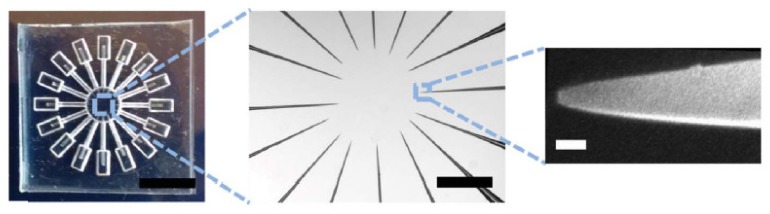
Characterizations of the multichannel device. (**left**) Macro image of PDMS-based device with 16 channels. (Scale bar = 8 mm.) (**middle**) Optical image of the center containing 16 glass nanopores at the tip of the capillaries, where a central chamber lies. (Scale bar = 500 μm.) (**right**) Scanning electron microscope (SEM) image of single glass nanopore on the tip of the capillary. (Scale bar = 100 nm.) Reprinted with permission from [39].

In the field of solid-state nanopore, the multichannel microfluidic system was first designed by Bell and coworkers with glass nanopores [39]. The glass nanopore was fabricated by drawing the quartz capillary down to a nanometer-scale aperture with a laser-assisted capillary puller, and the capillary was then melted from the middle and separated into two glass capillaries with a nanopores on the tip. Sixteen threadlike capillaries with a nanopore were then encapsulated into the PDMS and the tip of all these capillaries pointed to the central region of the electrolyte chamber as shown in Figure 4. A single grounded Ag/AgCl electrode was placed in the central region and sixteen active Ag/AgCl electrodes connected in the outer ring of the chamber so that sixteen different electrical signal paths were formed. A nanopores experiment with 12 electrodes was carried out, and three of the channels with the most pass events were counted. The results showed that their pass events all met the same hyperbolic distribution. This work also integrated a DNA origami-based squared nanopore on the tip of the glass nanopore, which extended the size of the nanopore system and improved the ability of precisely controlling of nanopore size. The capture of DNA origami across eight channels were monitored simultaneously under 600 mV. A characteristic step decreases in current occurred when one origami structure was a trap, and the baseline of ionic current could maintain a steady-state which satisfied the condition of nanopore detection.

**Figure 5:**
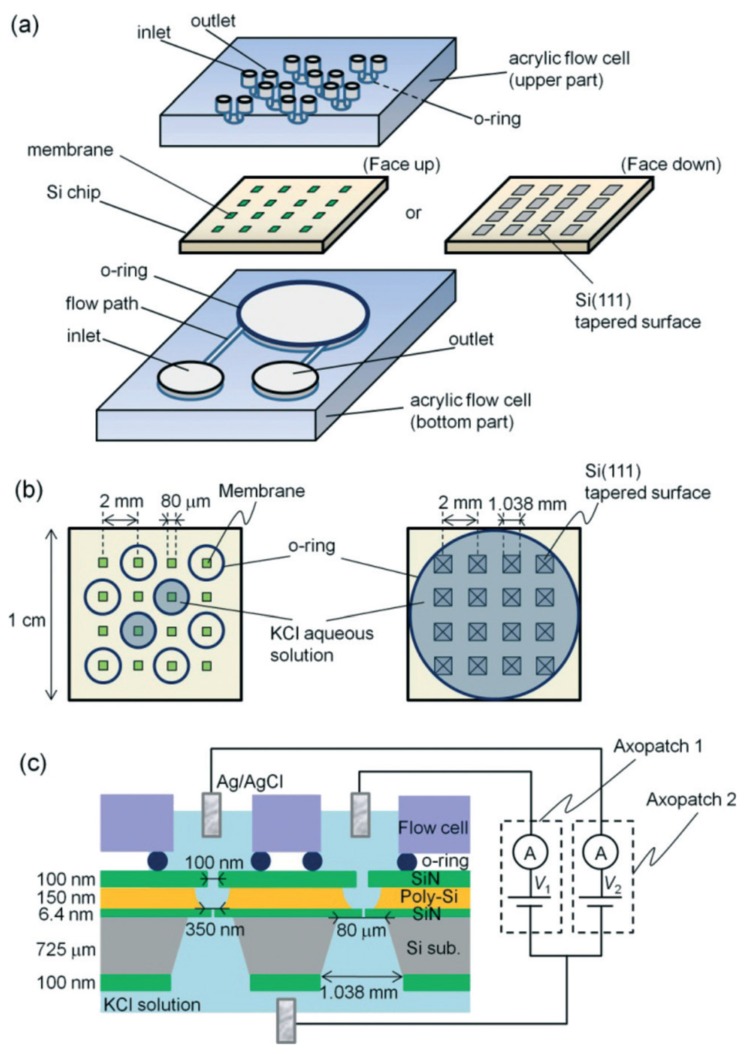
Structure of the two-nanopore measurement system. (**a**) Schematics of the flow cells and a membrane array chip. In the upper acrylic substrate, each two of the sixteen holes forms a collective; the middle nanopore chip is fabricated by standard micromachining process, forming an array of freestanding SiN_x_ membranes; in the bottom acrylic substrate, an o-ring surrounds a connected electrolyte chamber with inlet and outlet under the nanopore chip. (**b**) Schematic of the front and back sides of the membrane array chip. (**c**) Cross-sectional schematic of the chip and an equivalent circuit for a simultaneous measurement in the case of isolation happening on the front side of the chip. Reprinted with permission from [40].

Yanagi and coworkers first demonstrated the multichannel ionic-current detection system using SiN_x_ nanopores [40]. In their work, a 4 × 4 array of freestanding SiN_x_ membranes was formed on an 8-inch silicon wafer and on each membrane a nanopore was fabricated by multilevel pulse-voltage injection (Figure 5). This chip was then caught in the middle of two flow cells and the upper flow cell has eight individual chambers while the bottom flow cell forms one connected chamber. The author also found that when the chip was isolated from the back side (away from the side of the SiN_x_ membranes), a leakage current between the electrodes of two chambers was observed. This phenomenon was successfully suppressed by oxidizing the Si tapered surface. In order to further explain the influence of the oxide layer on the whole system, the open hole conductance before and after oxidation was measured. The mean value of conductivity in the oxidized chips (2.73 nS) is approximately half of that in the non-oxidized chips (5.56 nS), which could be attributed to the change of the thickness of the nanopore after the deposition of the oxide layer. From this point of view, this work not only proved the feasibility of multichannel detection by SiN_x_ nanopores, but also provided a solution to the possible leakage current problem in the parallel monitoring work on solid-state nanopores.

By equipping a special pair of electrodes to each nanopore channel, a multichannel detection device was successfully constructed. This combination can undeniably improve the speed of data collection and processing, and reduce the required concentration of analyte molecules. Such devices can also be more efficient in detecting a single sample under different conditions, such as nanopore size, bias voltage, and flow rate control.

### 3.2. Single Electrode for Multi-Channel Strategy

For a multi-electrode, multi-channel strategy, the detection throughput of nanopore sensors has been solved to some extent. While these devices required the electrode matches the channel one to one, which inevitably complicated and crowded spaces on the device. Furthermore, there is a limitation to the largest density of integrating two systems on the microfluidic chip, which means the maximum throughput of this strategy is totally restricted by the precision of micro-fabricating technology and the ability of collecting multichannel signals currently [41,42].

From an experimental point of view, there are other means to elevate the detection efficiency in addition to increasing the throughput. Generally, it is necessary for researchers to conduct several parallel control experiments to reveal the characteristics of analytes, such as the same analyte translocating nanopores with different sizes or different analytes translocating nanopores with the same size. The clogging of nanopores by analyte molecules is also unavoidable under such plenty of experiments. Hence, a nanopore–microfluidic system with the ability to carry out the above-mentioned experiments after one chip packaging process would greatly improve detection efficiency. In other words, multi-channel strategy is not for parallel nanopore detection, but for saving chip loading times and enriching the environment of detection.

**Figure 6:**
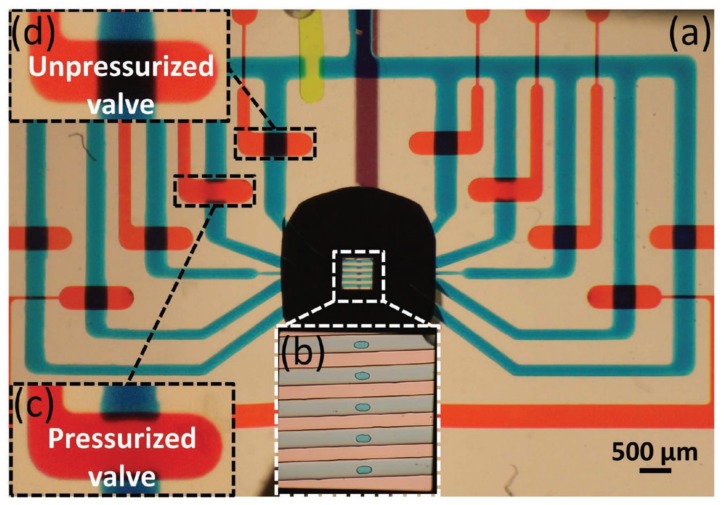
Optical image of the microfluidic system. (**a**) The overall picture of the integrated SiN_x_ chip in the microfluidic network, in which positioned the flow channels (blue), microvalves (red), routing valve (green) and a public bottom channel (purple). (**b**) Five flow channels cross over a 500 × 500 μm^2^ SiN_x_ membrane. (**c**) Pressurized status of the microchannel. (**d**) Unpressurized status of the microchannel. Reprinted with permission from [44].

Tahvildari and coworkers exquisitely combined controlled breakdown nanopore fabrication with the microfluidic system, which allowed the nanopores to be fabricated after the chip was encapsulated in the microfluidic system [43]. This combination of cheap nanopore fabrication method with nanopore detection system also lowered the threshold and increased the efficiency of the nanopore sensors’ preparation. They used a similar structure which one side connection and the other side isolation by five flow cells, but only one pair of electrodes were set in this system. Nanopores on different channels could only be prepared separately because a controlled breakdown was achieved by applying a pulse voltage to the ultrathin SiN_x_ film and breaking down its electrical defects. Their work demonstrated two strategies of nanopore fabrication, which were divided by different breakdown areas and showed various abilities to noise properties and single molecules detection. In their next work, they made some improvements to this structure by introducing microvalves to regulate the microfluidic and current pathways [44]. Five pairs of microvalves and flow cells were one-to-one corresponding relations so that every single pathway could be monitored individually. The structure of the microfluidic device is shown in Figure 6. A symmetric electric field and a looped flow channel were also conducted at the pore location to achieve a better nanopore fabrication and molecular sensing. More importantly, this work revealed that partially pressurizing microvalves can form a micro-environment in which the fluid is blocked while the current conducts. In their experiment, when the valve is unpressurized, the low-pass filter dominates the signal, while it is partially pressurized, the resistor-capacitor response dominates. This structure provided a wide operating space for the subsequent experimental design. Jain and coworkers reported a similar microfluidic structure in which the nanopores were fabricated before encapsulated into flow cells by using a focused ion beam [45]. Unlike the one-to-one controlled microvalves in Tahvildari’s work, they introduced four sets of microvalves and used the idea of logic gates to regulate eight different micro-channels. This design reduced the number of external microvalve channels and the complexity of the chip at the same time.

These works successfully improved the efficiency of nanopore experiments by introducing a single pair of electrodes to multi-channel microfluidic flow cells. For those experiments with a small sample size, these systems can provide different sized or functionalized nanopores to monitor the analyte molecules in a single sample-adding process. An identical experiment can also be directly conducted when a specific nanopore was clogged or its performance was decreased without reassembling of the microfluidic system. From the structural design point of view, this method does not have significant advantages over the first-mentioned method due to the need of microvalve control, but it can be improved by more sophisticated coding techniques.

**Figure 7:**
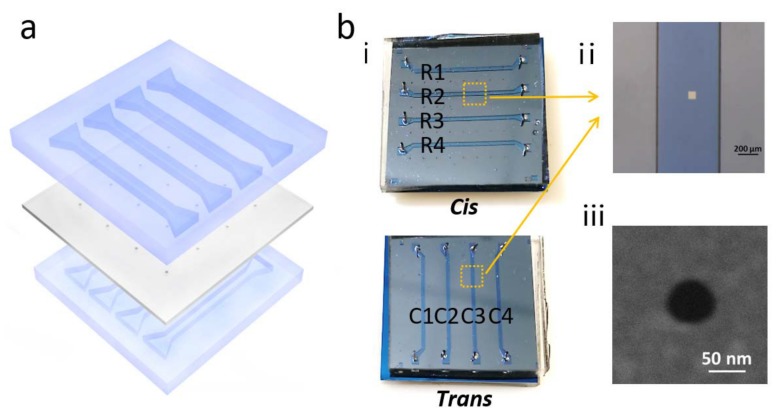
Schematic and photography of the integrated nanopore array microfluidic system. (**a**) Nanopore arrays with 8 × 8 nanopores on freestanding SiNx were fabricated by electron-beam lithography (EBL). The PDMS fluid channels with a width of 500 μm were produced by casting PDMS against a silicon wafer mold and then the PDMS was aligned and bonded on both sides of the Si chip in an orthogonal configuration. (**b**) (i) Optical images of the device after PDMS bonded on the nanopore chip, where only 4 × 4 nanopores were effective for electric conduction. (ii) Microscopic image of the freestanding SiNx area. (iii) SEM image of a 60 nm nanopore in the freestanding membrane. Reprinted with permission from [46].

Recently, Zeng and coworkers developed a nanopore devices which could individually address each nanopore on one array, by means of integrating microfluidic system and a multiplexer [46]. The setup of this device is shown in Figure 7. The multiplexer reduced the number of electrodes from sixteen pairs to only four pairs, and was able to address these nanopores in two modes: single-pore mode and multi-pore mode in selected row or column. When detecting a signal from one nanopore, because there was only one electrical pathway, no crosstalk would happen. While in the multi-pore detection, the ionic current would not be governed by one nanopore. In this work, potential control of each channel was strictly adopted to minimize the crosstalk. The drawback of this system is that although the acquisition signal can be switched as fast as possible among multiple nanopores, actually only one signal from one pore can be read out at the same time. However, the switch rate is unlikely to exceed the frequency which analyte molecules pass through the nanopores. Consequently, this device in its current state is helpful to reveal the crosstalk behavior of nanopores and fit with the theoretical electric field model, rather than directly applying it to multichannel parallel detection.

By adapting multichannel microfluidic design into nanopore sensors, the detection efficiency of nanopores has been significantly improved. While in terms of application and industrialization, which require high detection flux and portability of equipment, multi-electrode for multi-channel strategy just provides an experimental possibility for high-throughput DNA sequencing of nanopores. Due to the limited utilization of plane space on the chip, the improvement could be carried out from these two aspects: means of detection and full use of space. By changing the location of the detecting electrodes from both ends of nanochannel to closely placed around the pore, the in-plane current could be detected [47,48,49,50,51]. That might improve integration density for its lower crosstalk among adjacent nanopores compared to ionic current detection. Adapting other detection methods like optical detection into nanopore sensors is also a feasible scheme, which will be mentioned in the next section. The three-dimensional stacked scheme can make more efficient use of space, making the nanopore sensing device more portable. A single electrode for a multi-channel strategy is a way to improve the efficiency of experiments in the short run. However, taking the long view, it innovatively proposed an on-chip sample processing method, which would play a very important role in commercialized products.

## 4. Structure for Multifunctional Detection

Nanopore sensors are fascinating not only for its simple system and theory, but also for the enormous potential in integrating electrical detection with others means of methods. In the second generation DNA sequencing technology (next-generation DNA sequencing, NGS), the mainstream method is the detection and distinguishment of fluorescent-labeled base molecules [52,53,54,55], thus applying optical detection methods to nanopore sensors has become an important direction to solve the problem of DNA sequencing. However, optical detection has its own requirements, and several aspects are worthy of careful consideration when introducing it into nanopore sensors. Firstly, most optical detections are based on a total internal reflection fluorescence (TIRF) system or confocal imaging system [56]. For the TIRF system, an exponentially attenuated electromagnetic field forms near the interface, limiting the molecular excitation to this narrow region, resulting in a significant reduction in background light. For confocal imaging, the position in three dimensions should be precisely fixed to maximize the detection efficiency [57,58]. Both of these schemes have special requirements on the location of the nanopore chip. Especially in the TIRF system, the imaging interface is very close to the ultrathin nanopore membrane while the working distance of objective with high numerical aperture is very small, which leads to the difficulty of solution chamber fabricating. Secondly, fluorescence groups have the problem of quenching and gathering. The concentration and movement of analyte molecules with fluorescence in the electrolyte should be properly controlled. Hence, it is necessary to provide a better optical detection environment by means of the integration of microfluidic flow cells to nanopore sensors.

### 4.1. Optical Sensing for Parallel Detection

As mentioned in the previous section, simply increasing the number of channels and electrodes is very limited to improving the detection flux of nanopores. Electrical signal crosstalk among different nanopores can only be eliminated by physical isolation. However, for optical signals, as long as the distance between the two nanopores is greater than the limit resolution of the optical platform, the optical signals passing through different nanopores will not have a crosstalk problem, which makes optical sensing a promising way for parallel detection of solid-state nanopores.

**Figure 8:**
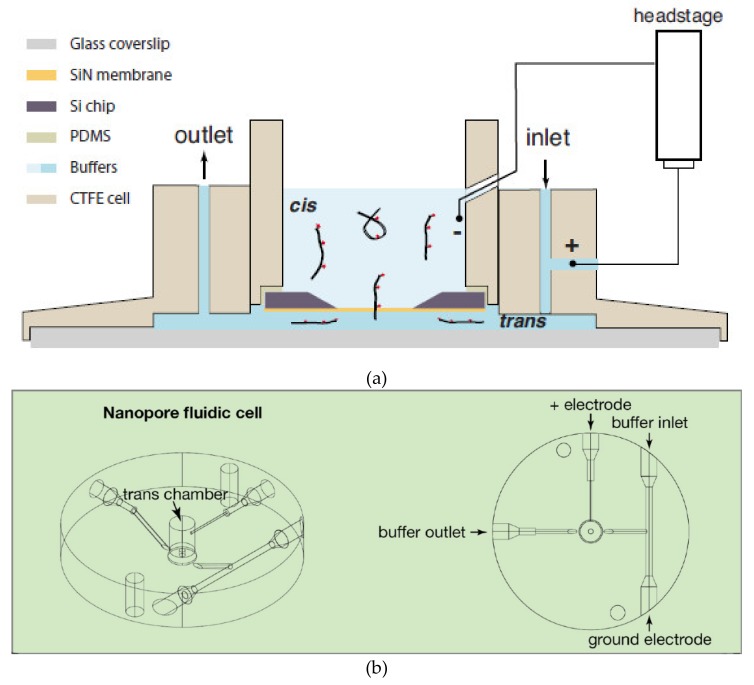
Structure of the microfluidics of the nanopore optical detection systems. (**a**) Schematic illustration of the fluorescence-labeled nanopore sensor. Thin layers of fast curing PDMS are used to glue the Si chip and the glass slip to the insert and outer cell. Ionic current through the nanopore is monitored using a pair of electrodes immersed in the cis and trans chambers. (**b**) Schematic of the label-free nanopore sensor. The nanopore chip is located in the middle of the microfluidic chamber and the bottom of the flow cell is thin enough for a total internal reflection fluorescence (TIRF) detection. The upper of the chip, on the channel for inlet and outlet, which represented by a liquid chamber. Reprinted with permission from [59,65].

Two alternative methods have been put forward to realize this exciting idea. Detection of fluorescence-labeled DNA using solid-state nanopore was first proposed by Soni and coworkers in 2010 [59]. The structure of the microfluidic system involved in the work is shown in Figure 8a. The whole system was placed on an ultrathin glass coverslip and thin layers of fast curing PDMS were used to glue every part of the system, including silicon chip with a nanopore, glass coverslip and insert and outer cell. DNA and DNA-protein complexes were successfully observed by this TIRF system with a temporal resolution of ~1 ms. The potential in DNA sequencing of this system was then tested by an array of nanopores and two-color barcoding DNA sequences [60]. The performance was further enhanced by the reduction of the background photoluminescence of SiN_x_ membranes by e-beam irradiation [61]. The main characteristic of this method is the direct fluorescent labeling of analyte molecules, and the read signals directly reveal the different components and shapes of analyte molecules. However, the introduction of fluorescent molecules changes the original molecule state and molecule configuration, which may affect the fidelity of signals. The binding efficiency of fluorescent molecules and analyte molecules is also worth considering when decoding the fluorescent signals.

Recently, a method for monitoring ion flow through nano-channels was applied to solid-state nanopore sensors, in which Ca^2+^ sensitive fluorescence was used as indicators of changes in ionic flow [62,63,64]. When analyte molecules translocated through the nanopore, the concentration of ion in nanopore was decreased consequently and this state would be revealed by the reduced fluorescent intensity. Ivankin and coworkers utilized this fluorescence label-free method to parallel solid-state nanopore ionic current measurements [65]. In their study, a circular two-layer microfluidic system was set on the ultrathin glass slide and the fluorescence signal was acquired by a 60× objective with the numerical aperture of 1.49 in TIRF optical system, which is shown in Figure 8b. It is worth mentioning that the signals were monitored at a quite low concentration of Ca^2+^ in this study compared with conventional nanopore detection systems, which indicted huge potential in applying to DNA sequencing with specific enzyme environment and improving SNR by increasing ionic concentration. Compare with fluorescence-labeled method, direct observation of ion flux showed the advantage of simple operating steps and high fidelity. However, currently, the temporal resolution of both methods is fairly low, and they are more suitable as a complement to the electrical detection method.

In conclusion, applying optical detection methods to nanopore sensors showed a great performance in increasing the throughput of nanopore sensors. Although the temporal and spatial resolution are not up to the standard of DNA sequencing, the optical detection method shows promising prospects because of the rapid development of the optical detection platform. Structurally, the microfluidic system with an ultrathin channel plane and a nanopore chip with fixed position are necessary to ensure an appropriate environment for optical detection. With the continuous progress of micro-nano manufacturing technology, ultrathin channel structure and nanoscale precision will no longer be the barrier to build the optical detection platform of nanopores.

### 4.2. Direct Observation of Analytes

DNA sequencing, which is devoted to single-base resolution and high throughput, is one of the most important applications of nanopore technology, but it is not the only direction of DNA molecule analysis. Direct observation of fluorescence-labeled DNA molecules is a good way to characterize these molecules, which has been successfully optimized in the genome mapping technique (Bionano) [66,67]. This method has become an auxiliary means for assembling the contigs and scaffolds of second-generation sequences. Moreover, visualization of the motion of DNA molecules would provide experimental information to assist the theoretical analysis of DNA electrophoretic and electroosmotic characteristics under the test environment of nanopores.

Ando and coworkers directly observed the motion of YOYO-1 stained DNA molecules near the nanopore by a home-built nanopore-microfluidic system, and determined that the electrophoretic driving force exceeds the thermal fluctuation force [68]. In their setup, the silicon chip with nanopore was tightly placed on a coverslip and the intermediate zone was filled with solution. Similarly, Kurz and coworkers visualized the motion of fluorescent DNA at a very dilute concentration using a confocal microscope [69]. To make sure the operation environment, the microfluidic system is mounted on the piezo-stage, which has nanometer-scale precision along three dimensions.

**Figure 9:**
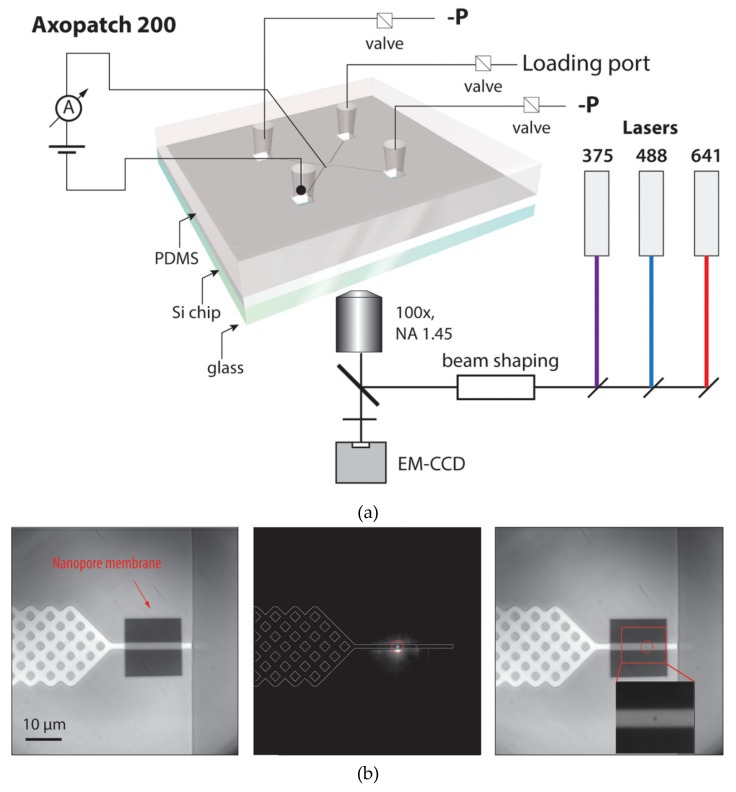
Schematic and photographs of the electro-optical detection system. (**a**) A PDMS-Si-glass three-layer structure is adopted in the device, in which Si chip is anodically bonded to glass and plasma bonded to PDMS. A laser generator with 488 nm and 641 nm excitation light and 376 nm laser to nanopore fabrication is employed. There are four liquid reservoirs made in the PDMS layer on the backside of the device, which enables the application of negative pressure to control the motion of analyte molecules. A freestanding SiN_x_ is located at the center of the chip and analyte buffer can be loaded on the glass side. (**b**) The microgram of the microfluidic channels leads to the nanopore area and the process of laser-drilled nanopore. An interfaced pillar array is used to uncoil the ultralong genomic DNA. Reprinted with permission from [70].

Just recently, Zrehen and coworkers designed a nanopore integrated microfluidic device for electro-optical detection of ultralong human genomic DNA [70]. The schematic illustration of this device is shown in Figure 9a. For high magnification multicolor single-molecule fluorescent imaging, the system was fabricated by a complete in-silicon design and permanently fused to borosilicate glass, which was only 150 μm. In order to achieve accurate position and size of nanopores preparation after chip packaging, a laser drilling method was also adopted [71,72], which avoided the damage of nanopore caused by the high temperature and stresses during anodic bonding. The interfaced pillar array (Figure 9b) successfully uncoiled the ultralong DNA molecules before they enter the nanopore. Through the collaboration of optical observation and pressure control, specific DNA molecules can be passed through the nanopores and simultaneously detected electrically and optically.

Structures of the microfluidic system applied in these works also showed the necessity of the ultrathin channels between the optical detector and the membrane with nanopores. Fortunately, the other parts of this system, such as the microfluidic channels on the upper side and the electrodes, can be simplified thanks to the absence of real-time ionic current detection in some devices. The findings of the motion of DNA molecules around nanopores will be great helps to understand the mechanism of biomolecules’ motions in the nanopore region, including the effect of electrophoresis and electroosmosis, the interactions among biomolecules, wall of nanopores and ultrathin substrate membrane.

### 4.3. Optofluidic Integrated Nanopores

Optofluidic devices, which integrate the functions of light generation, control and processing into one microfluidic system, have been applied in many fields, such as cytobiology, analytical chemistry and microtechnique. With the addition of nanopores, optofluidic devices equip the ability of single-molecule detection. The flexible location of light detection in optofluidic also provides more possibilities for the design of experimental devices.

**Figure 10:**
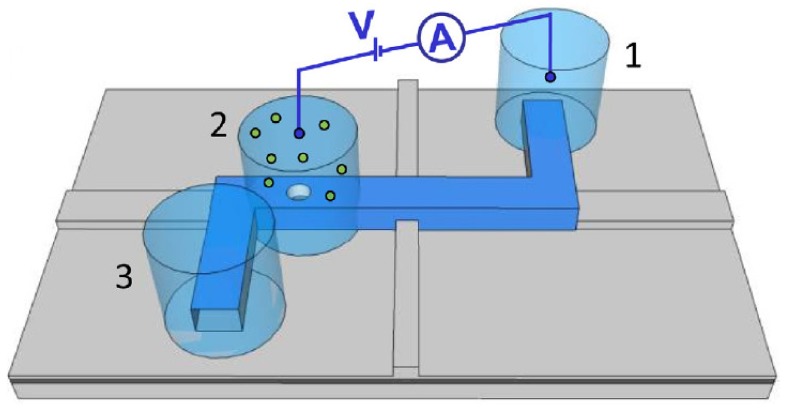
Structure of the microfluidic system with an integrated solid-state nanopore. The zigzag microfluidic channel is shown in blue. Three reservoirs are set as the buffer and analyte introduction channels. Nanopore chip is mounted at the middle reservoir, where the only channel for analyte molecules from upper to the bottom chamber. The excitation light is applied from the vertical in the picture and the light detector is set horizontally. Reprinted with permission from [75].

Liu and coworkers developed an optofluidic chip to correlated electrical and optical analysis and successfully detected H1N1 influenza A virus and λ-DNA with optical sensitivity to individual fluorescently labeled molecules [73,74,75]. The structure of this microfluidic system is very delicate, as shown in Figure 10. Three fluidic reservoirs are used for buffer and analyte introduction into the channel, with the middle one having a nanopore connecting the upper and bottom solutions. The excitation laser and detector are orthometric at the bottom flow cell. When analytes are driven by electrophoretic, they translocate through the nanopore and excited by the laser. The signal is monitored optically and electrically at the same time.

Without the need for TIRF or confocal imaging systems, it is easier to fabricate the whole detection device. On the contrary, the low requirement of optical detection equipment also reduces the limit resolution of the detection system. Compared with DNA sequencing, such an optofluidic platform is more suitable for single-molecule counting, molecular recognition and screening.

To sum up, the introduction of an optical detection device to a microfluidic integrated nanopore system provides a variety of ways for assisting single-molecule detection. By marking analytes with fluorescent molecules, parallel detection of mass nanopores or even direct observation of the analyte state through a single pore can be realized. However, the problem introduced by this method is that the original state of analytes cannot be restored. Non-labeled fluorescence method overcomes this shortcoming to some extent, but its stability in long-term detection cannot be guaranteed due to the precise setting of fluorescence gradient, which is closely related to the detection accuracy of fluorescence signal. As with the NGS, the stability and reliability of fluorescent molecules is also a matter worthy of general consideration. The optofluidic system is more like an indirect way to obtain signals. Instead of focusing on the nanopore, the detection focuses on the observation of the state in the fluid pipeline. Furthermore, the optical structure makes it particularly difficult to detect details inside individual molecules, such as the sequencing information of DNA or protein molecules. However, back to the problem of parallel detection of nanopores, the introduction of optical means is still a more promising method compared with multi-electrode integration. This conclusion can be drawn from two aspects. On one hand, the specificity and stability of the fluorescent probes have been improved by different fluorescent groups and combination strategies [76,77,78]. On the other hand, there is still room for improvement of photosensitive devices. In particular, the application of 3D stacked chip technology to photosensitive array chips will significantly improve the accuracy and speed of optical signal processing [79].

## 5. More Integrated Nanopore-Microfluidic System

Nanopore sensing requires the cooperation of multiple steps, including sample preparation, purification, detection and data analysis. Taking nanopore sensing of protein molecules as an example: the first step is the preparation of the sample, in which different molecular purification processes are involved; the next step is the loading of the sample, and the surface characteristics of the microchannel is mainly concerned as well as the viscosity of electrolyte buffer; the following comes to the detection of samples, in which pH and temperature of the system should be carefully controlled to provide an optimum environment for protein molecules translocation. After all these processes conducted, ionic current signals are detected and processed for characterizing protein molecules. However, the common microfluidic systems integrated on nanopore sensors are mainly focused on parts of these processes, such as sample loading and detection. This incomplete design can indeed provide a stable experimental environment for specific experiments, but its unicity and non-extensibility are also obvious. Therefore, a highly integrated nanopore detection system is very necessary, especially for rapid diagnosis, outdoor diagnosis and other practical applications.

**Figure 11:**
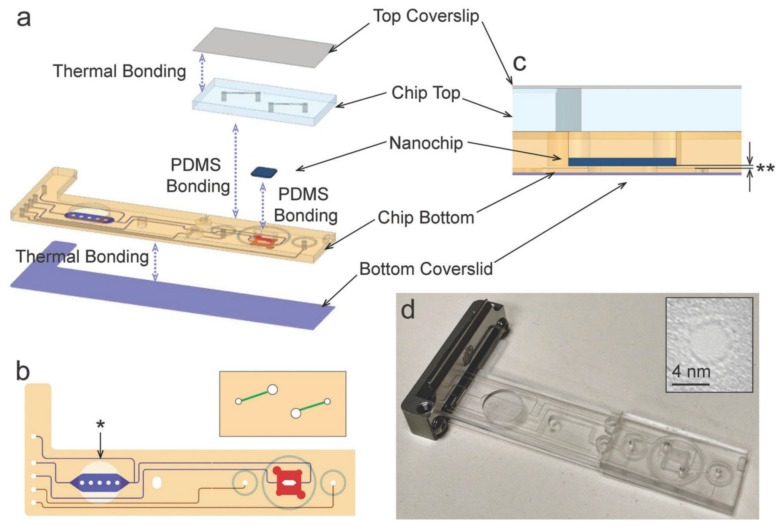
(**a**) Schematic of the microfluidic chip assembly. (**b**) Top view of the chip. The chip made by Zeonex plastic can be divided into the top part and bottom part. On the bottom part, the channel leading to or from the sample preparation chamber and bottom of nanopore chip are labeled blue, while the channel leading to or from the top of nanopore chip are labeled red. On the top part, the green channels connecting the red channels and the top of nanopore chip. The top chip is sealed with PDMS glue to the bottom chip using the 3 micromachined tracks in the bottom chip as a guide. (**c**) Cross-section of the nanopore chip in the chamber. For potential optical detection applications, the chip bottom is fabricated from both sides to achieve the thinnest layer of 200 μm. (**d**) Photograph of the microfluidic chip after assembling, and the inset shows the nanopore loaded on. Reprinted with permission from [80].

Lately, Varongchayakul and coworkers demonstrated an integrated stand-alone solid-state nanopore-microfluidic chip, which achieved the highest integration degree ever reported [80]. The structure of the microfluidic chip assembly is shown in Figure 11. In their structure, a reaction environment at room temperature up to 75 °C could be achieved by an accurate temperature control unit. An external processing system that can handle up to eight different electrolyte solutions was also integrated into the nanopore detection system, which allowed the fast change of the solution environment. The microfluidic system also had room for DNA purification, allowing polymerase chain reaction (PCR) products of DNA molecules to be fed into the chip and purified by magnetic beads before the DNA molecules reach the trans chamber of the nanopore chip. This system with the compatibility of magnetic beads can also be applied to the separation of an analyte from a mixed biological environment. What’s more, the integrated system had the potential of simultaneous electrical and optical detection as long as the bottom microfluidic channels are optimized.

To verify the reliability of this device, electrical integrity and noise analysis were performed firstly compared with the conventional setup made of Teflon. Two nanopores of almost the same size (5 nm) were loaded into conventional setup and microfluidic setup, respectively, and the same electric field of 500 mV was applied to them. The results showed that the root-mean-square noise value in conventional setup was about 185 pA, while that in microfluidic setup was about 192 pA, indicating that the integrated nanopores did not significantly increase the noise of the detection equipment. At the same time, the low 1/f noise of the two setups also maintained the same level. Then, the performance of the device was evaluated by the 5 kbps-long DNA translocation experiments, and the results were also consistent with that in the conventional setup with different pore sizes. Finally, the utility of the device to purify crude PCR products was conducted by the synergistic effect of the magnetic field unit and temperature control unit. The translocation events were bimodal distribution located at 0.51 ± 0.20 and 0.83 ± 0.20 in amplitude and single distribution located at 125 ± 18 μs in time, which were similar to that of NoLimits ^TM^ DNA in the same purification buffer.

More integrated nanopore-microfluidic system achieved better experimental results and presented more advanced design concepts compared with conventional setups, which is particularly evident under the increasingly complexity of detecting conditions. It is certain that high integration with sample preparation, loading and detection will be the final shape of the nanopore-based microfluidic systems. Meanwhile, the capacity of mass production and the cost and difficulty of manufacturing determine its potential of commercialize.

## 6. Conclusions and Outlook

Integrating microfluidic with nanopore sensors has been evolving rapidly in the past two decades, bringing more possibilities to single-molecule detection and other nanopore-based applications. This combination of these two elements is a natural but huge-potential method. In this review, we focused on the specific microfluidic structures in different applications, which would provide references for the readers with the requirements of microfluidic structure design in the future.

Microfluidic systems integrated in nanopore sensors are artful and varied. In the early experiments of nanopore detection, the microfluidic system has been playing the role of fixture and has no further influence on the nanopore detection. However, the increasingly high detection requirements, such as accuracy, flux and detection method, have made microfluidic become an indispensable part of the nanopores detection system. In order to improve the flux of nanopore detection, multichannel nanopore detection microfluidic systems are designed. Ultrathin microfluidic systems with ultrathin flow channels are also developed due to the requirements of nanopore optical detection. Highly integrated microfluidic systems are finally invented to adapt to more complex nanopore detection conditions. Ultimately, microfluidic structures are designed to make it easier for nanopore detection.

Accordingly, due to the introduction of a more integrated and complex microfluidic system, the preparation method of nanopores has to be changed. In the beginning, all nanopores were fabricated before the microfluidic system was bonded to the nanopore chip by means of focused ion beam (FIB) or focused electron beam (FEB). However, although nanopores fabricated by these processes are easier to characterize and have better homogeneity, the preparation cycle is long and expensive, which cannot meet the demand for high throughput nanopore detection. This led to the generation of bulk fabrication methods of nanopores, such as electron beam lithography. In addition, due to the limitation of the microfluidic integration process, which may need high temperature or pressure resulting in fragile membranes broken, the demand of bonding before nanopore preparation is generated. Therefore, it is necessary to introduce a controlled dielectric breakdown method into the microfluidic system. Controlled dielectric breakdown technology is very sensitive to the state of the ultrathin film, so it reversely promotes the refinement of the microfluidic device. Besides, the rise of optical detection of nanopores has led to the application of laser etching technology in the fabrication of nanopores. In a word, the preparation of nanopores and the design of microfluidic systems need and grow mutually, and this virtuous cycle will push the whole nanopores sensing forward.

However, it must be admitted that the integration of solid-state nanopores is still not up to the requirements, and the transformation from laboratory setups to clinical used devices is still a long way off. According to the current development situation, the integrated nanopore-microfluidic system is developing towards two polarizations, one is to pursue the ultimate simplicity, and the other is to move toward commercialization. For extremely simplified designs, the most important design concept is the protection of the original electrolyte solution environment. For a multichannel system, parallel detection may encounter a bottleneck, because the theoretical space utilization is capped, and the current micro-nano processing technology is difficult to reach and overcome this limit. The method mediated by multiplexer has little effect on reducing the complexity of the space on the nanopore chip and the signal crosstalk among different nanopores. However, the good news is that the preparation and manipulating of samples in multichannel can be a promising export for this structure. From the perspective of experimental operation, this method of reducing the labor of the experimenter is very effective. For solid-state nanopore sensors, optical detection is the most likely method to achieve high throughput analysis with further improvements of the optical signal-to-noise ratio (SNR).

## Abbreviations

DNA	deoxyribonucleic acid
PET	polyethylene terephthalate
SiN_x_	silicon nitride
PDMS	polydimethylsiloxane
PEG	polyethylene glycol
TIRF	total internal reflection fluorescent
SNR	signal-to-noise ratio
PCR	polymerase chain reaction
FIB	focus ion beam
TEM	transmission electron microscope (microscopy)
SEM	scanning electron microscope (microscopy)
NGS	next-generation sequencing
EBL	electron beam lithography
FEB	focus electron beam
